# Open-Set Recognition of Human Activities from Head-Mounted Inertial Sensor

**DOI:** 10.3390/s26031079

**Published:** 2026-02-06

**Authors:** Angela Cortese, Sarah Solbiati, Alice Scandelli, Andrea Giudici, Niccolò Antonello, Diana Trojaniello, Giacomo Boracchi, Enrico Gianluca Caiani

**Affiliations:** 1 Department of Electronics, Information and Bioengineering, Politecnico di Milano, Piazza Leonardo da Vinci 32, 20133 Milan, Italy; angela.cortese@polimi.it (A.C.); sarah.solbiati@polimi.it (S.S.); alice.scandelli@polimi.it (A.S.); andrea.giudici@polimi.it (A.G.); giacomo.boracchi@polimi.it (G.B.); 2 EssilorLuxottica Smart Eyewear Lab, EssilorLuxottica, Piazzale Luigi Cadorna 3, 20123 Milan, Italy; niccolo.antonello@luxottica.com (N.A.); diana.trojaniello@luxottica.com (D.T.); 3 IRCCS Istituto Auxologico Italiano, Via Ludovico Ariosto 13, 20145 Milan, Italy

**Keywords:** human activity recognition, inertial measurement units, wearable sensors, smart eyewear, open-set recognition

## Abstract

**Highlights:**

**What are the main findings?**

- Existing head-mounted human activity recognition (HAR) classifiers can be extended to detect previously unseen activities by adding lightweight, post hoc open-set mechanisms without modifying the model architecture or retraining procedure.
- A rigorous leave-one-activity-out evaluation shows that effective unknown activity detection can be achieved from head-mounted inertial data using low-complexity open-set scoring methods.

**What are the implications of the main findings?**

- The lightweight nature of the explored techniques indicates their potential compatibility with resource-constrained wearable platforms.
- The empirical findings provide practical insights for designing real-world open-set HAR systems, particularly regarding dataset composition.

**Abstract:**

Human activity recognition (HAR) based on inertial measurement units (IMUs) embedded in wearable devices has gained increasing relevance in healthcare, wellness, and fitness monitoring. However, most existing classification methods assume a closed-set setting, where all activity classes need to be defined during training, which limits their applicability in real-world environments where unseen or unexpected activities are present. To overcome this limitation, we adopt an open-set recognition (OSR) framework that requires minimal changes to the HAR classifiers traditionally employed for this purpose. We also provide an extensive empirical evaluation based on a leave-one-activity-out validation protocol applied to two datasets with IMU signals acquired from smart eyewear: a proprietary dataset and the publicly available UCA-EHAR dataset. A lightweight one-dimensional convolutional neural network was trained to classify six-axis IMU data across common activities. We assess open-set HAR performance using several methods requiring limited computational overhead and operating in the logit space, including maximum logit, Gaussian Mixture Models, Kernel Density Estimation, OpenMax, and Nearest Neighbor Distance Ratio. Robust identification of unknown activities was achieved, with area under the ROC curve > 0.8. These findings highlight the potential of low-complexity open-set approaches for real-time HAR on resource-constrained wearable platforms, supporting the development of adaptive and reliable sensor-based recognition systems for real-world use.

## 1. Introduction

Human activity recognition (HAR) consists in the automatic identification of human actions, enabling a wide range of applications in activity monitoring, fitness tracking, healthcare, and human–computer interaction [1]. Existing HAR approaches can be broadly categorized into camera-based or wearable sensor-based systems. While the former can achieve high accuracy, these solutions raise critical challenges related to privacy, environmental constraints, and computational costs [2]. In contrast, the latter, by employing inertial measurement units (IMUs), offers a practical alternative due to low cost, portability, energy efficiency, and potential ability to support continuous monitoring in daily life [3]. Many studies in sensor-based HAR have previously focused on smartphones and wrist devices (e.g., smartwatches) that provide rich motion data in a convenient manner. IMUs embedded in head-mounted devices, such as earbuds or smart eyewear, have been less explored in the literature, despite offering a very promising sensing modality, being capable of capturing motion patterns that are more directly linked to the head and upper-body activities.

Established methods for HAR span from classical hand-crafted feature extraction and machine learning (ML) approaches to end-to-end deep learning (DL) models [4], achieving overall great accuracy. HAR systems are generally designed under a closed-set assumption where the set of activities to be classified is already fixed and fully known during the training phase. In real-world scenarios, this assumption is rarely met. Users may perform activities not originally included in the training set, either because the set of target activities was incomplete or because entirely new behaviors could emerge over time. Such an open-set condition would require classifiers to recognize not only known activities but also samples belonging to previously unseen classes. The open-set recognition (OSR) problem has been largely investigated for image classification [5], but less effort has been made in the context of time-series analysis for HAR [6].

Despite OSR having been widely investigated in computer vision, OSR remains an unexplored problem in IMU-based human activity recognition. HAR recognition models are trained to process multivariate temporal signals characterized by strong inter-axis correlations, pronounced inter-subject variability, and high overlap between activity classes. Activities such as walking, running, and stair climbing often share similar motion patterns over short temporal windows, resulting in less separable representations. These characteristics make OSR behavior in IMU-HAR nontrivial and worth exploring.

Two main challenges characterize the application of OSR methods applied to HAR from wearables: (i) the model must be trained without explicit samples from the unknown classes, making it necessary to detect novelty by a classifier trained on the distribution of known classes; and (ii) OSR solutions must remain computationally efficient to be deployable on resource-constrained platforms. Many existing OSR approaches, such as adversarial learning or specialized architectures [5], offer strong performance but require complex training pipelines or additional model parameters, which can hinder deployment in embedded systems.

To overcome this limitation, we present an open-set HAR framework for head-mounted inertial data acquired through smart eyewear. The proposed approach leverages neural classifiers trained on raw time series from a multichannel IMU and addresses OSR through lightweight, post hoc scoring strategies applied to the classifier outputs. Temporal dynamics and inter-axis relationships are modelled at the representation level by a convolutional backbone, while open-set performance is systematically evaluated under identical training conditions through a sound validation protocol. This formulation enables a comprehensive assessment of the robustness and suitability of different lightweight OSR methods for wearable HAR scenarios.

## 2. Related Works

This section reviews prior works relevant to our study. We first summarize HAR methods based on head-mounted IMU sensors (Section 2.1), provide an overview of OSR techniques (Section 2.2), and finally discuss existing open-set approaches applied to wearable devices (Section 2.3).

### 2.1. HAR from Head-Mounted IMU

There is a large body of literature investigating the use of IMUs for HAR. The rise of DL, supported by large, labelled datasets, has transformed HAR, moving from relying on hand-crafted features to end-to-end representation learning with deeper architectures, thus increasing the performance [4]. Although head-mounted IMUs are less common in HAR, they provide access to subtle head motion patterns that can be informative for recognizing human activities. Their adoption has been facilitated by the widespread availability of Virtual Reality (VR) headsets, smart earplugs, and smart eyewear. In this context, head-mounted IMU systems typically target everyday activities such as sitting, walking, stair climbing, and running.

Recent work focused on ear-worn sensors showed that traditional ML approaches and DL models (e.g., DeepConvLSTM, ConvTransformer) can classify such daily activities with competitive performance [7]. Smart glasses have emerged as another promising platform for collecting IMU data for HAR, and both DL and ML methods have been explored in this domain application. For example, Stankoski et al. [8] investigated the use of the multi-sensor OCOsenseTM smart glasses to recognize everyday activities, evaluating three end-to-end DL architectures that showed promising results. Similarly, Ho et al. [9] compared the classification performance of a Support Vector Machine (SVM) with IMU data collected either from a smartphone or smart glasses across four activities. One of the most recent contributions in this field is the UCA-EHAR dataset [10], which has been used to train recurrent neural networks (RNNs) for activity classification and to analyze power consumption during live inference on the microcontroller of smart glasses. The UCA-EHAR dataset has also been employed in other studies; for example, Mekruksavanich et al. [11] applied a Convolutional Neural Network with Long Short-Term Memory (CNN-LSTM) hybrid architecture, where convolutional layers extract spatial features form IMU signals and LSTM units model their temporal dynamics, reaching an F1-score of 93%. More recently, Sloan et al. [12] explored UCA-EHAR within a cross-body transfer learning framework, showing that pre-training models on data collected from other body locations can improve activity classification on the head-mounted IMU by up to 8% in accuracy.

Despite these advances, limited effort has been devoted to detecting activities performed outside the set of actions represented in the training data. In fact, HAR is typically framed as a closed-set classification problem, where the model is restricted to classify among the known classes it was trained on. In practice, however, users may perform new or unforeseen activities, making it infeasible to anticipate and label all possible scenarios. This challenge, known as the OSR problem, has been widely investigated in the image classification domain and, more recently, in HAR time-series data [13,14,15,16,17,18,19]. Nevertheless, to date, these approaches remain unexplored in the context of HAR from head-mounted IMUs.

### 2.2. Open-Set Recognition

The concept of OSR was formally introduced by Scheirer et al. [20], who defined the open space risk and highlighted the need for classifiers to detect samples from classes not observed during training, in contrast to traditional closed-set recognition, where the classifier is only requested to identify labels occurring in the training set. This foundational work established OSR as a distinct research problem, emphasizing the balance between correct classification of known classes and reliable rejection of unknowns. Building on this formulation, Bendale and Boult [21] proposed OpenMax, the first DL-based method for OSR, which adapts the SoftMax layer to allocate probability mass to an explicit “unknown” class.

A common baseline approach relies on the SoftMax confidence score to identify samples as out-of-distribution (OOD) [22]. However, SoftMax outputs can lead to overconfident predictions for inputs far away from the in-distribution data [23], such as data belonging to classes never seen during training. Dai et al. [24] showed that the logit vector (i.e., the raw output of the final network layer before SoftMax) provides more reliable OOD indicators, with the maximum logit value often used as a simple yet effective OSR score. Similarly, Liu et al. [25] demonstrated that the energy score, derived from the logit outputs, outperforms traditional SoftMax-based methods. Other techniques, such as temperature scaling [26,27] and entropy regularization [28], have been proposed to mitigate the classifier overconfidence.

Beyond discriminative approaches, generative and instance generation approaches have also been explored. These methods explicitly model the data distribution of known classes in order to detect outliers. Techniques based on Generative Adversarial Networks (GANs) and Variational Autoencoders (VAEs) are particularly prominent in this area. GAN-based approaches generate realistic samples that capture the intrinsic variability in known categories, while VAE-based methods learn class-conditioned latent representations that help characterize the boundary of known classes, enabling reconstruction- or likelihood-based rejection of unknown samples in and open-set setting [29,30,31].

All these advances were developed in the image recognition context, and the transfer of OSR methodologies to wearable sensor modalities and, in particular, to IMU data for HAR remains largely underexplored.

### 2.3. Open-Set Recognition from Wearable Sensors

In distance-based and metric learning approaches, a representative contribution is the Mix-up Triplet Mahalanobis Distance method by Lee and Kim [13], which employs augmented triplets with stochastic mix-up to learn discriminative embeddings, and applies Mahalanobis distance thresholds for unknown activity rejection. While effective, this approach requires specialized training loss and embedding space design, which limits comparability across datasets and architectures. Similarly, Lima et al. [14] proposed NOvelty discrete data stream for Human Activity Recognition, an online novelty detector for smartphone HAR based on symbolic discretization and histogram comparison. Although computationally efficient, its symbolic representation may fail to capture fine-grained temporal dynamics that are essential in wearable HAR. Both [13,14] are certainly less practical than OSR solutions that do not require training and operate on a HAR classifier trained in standard settings, like the one we considered in our OSR framework for HAR.

Roy et al. [15] introduced Deep Time Ensembles, which estimate uncertainty by aggregating predictions across models trained on different temporal windows. This approach was later extended to fall detection, treating falls as out-of-distribution events [16]. These works highlighted the utility of uncertainty modelling in OSR; however, ensemble-based methods introduce significant computational overhead and may lack scalability in real-time HAR systems mounted on a wearable device.

Self-supervised and contrastive learning methods were also investigated in HAR. Kim and Lee [17] developed CLAN, a self-supervised framework for new activity detection that integrates contrastive learning with customized augmentations and time-frequency representations. This design achieves strong performance on different datasets but requires dedicated self-supervised pre-training and data augmentation pipelines, which are not straightforward to employ.

Finally, Boyer et al. [18] presented one of the first systematic evaluations of OOD detection methods for HAR from smartwatch IMUs, comparing shallow ML baselines, deep feature approaches, and post hoc scoring rules such as Softmax thresholding, Out-of-DIstribution detector for Neural networks (ODIN), and OpenMax. Their findings suggest that simple methods can perform competitively compared to more complex deep models, highlighting the importance of computational efficiency. However, these solutions were not investigated in the context of HAR using recordings from eyewear-mounted IMUs.

In addition to segment-level classification and rejection strategies, a complementary line of work focuses on modeling human behavior at the level of activity sequences and routines. Process-oriented approaches, such as the workflow-based incremental learning framework proposed by De Carolis et al. [19], represent user behavior as evolving activity structures that can be refined over time when new activities or transitions appear. While this paradigm is typically explored in ambient-sensing smart environments and does not frame the problem as open-set recognition, it highlights an important perspective that is also relevant for wearable IMU-based HAR: the presence of previously unseen activities may emerge not only as an isolated segment that does not match any known class but also as a systematic inconsistency at the routine level. In our context, this perspective highlights that unknown activities may manifest not only at the level of individual IMU segments but also as inconsistencies with respect to habitual activity sequences.

## 3. Materials and Methods

### 3.1. Datasets and Sensor Setup

Inertial data were collected (sampling frequency 100 Hz) using a 6-axis IMU sensor (three-axis accelerometer and three-axis gyroscope) embedded in an eyewear frame prototype. The sensor was positioned on the left temple arm of the glasses; further hardware specifications and implementation details of the utilized smart eyewear platform can be found in [32]. Two experimental protocols were designed: static and dynamic. In the former protocol, thirty healthy volunteers (15 M and 15 F, age median [25th percentile; 75th percentile] 27 [24.3; 32.3] years) were recruited to perform in a seated position a core set of head-related activities, including *chewing*, *drinking*, *nodding*, *shaking* and *breathing*. In the dynamic protocol, a different group of ten subjects (5 M and 5 F, age 27.5 [24.3; 31.5] years), performed other activities, such as *speaking*, *walking*, *stair climbing*, and *cycling*. All recordings were carried out in a controlled laboratory environment. Each activity duration varied between 1 and 5 min; details on the duration of each activity per subject in the static and dynamic protocols are reported in Table 1 and Table 2, respectively. Specific instructions on how to perform each designated activity were given to participants, with no constraints to artificially restrict or modify their natural movements.

All participants provided written informed consent prior to enrollment. The study procedures were conducted in accordance with the Declaration of Helsinki (1975, revised in 2013), and ethical approval was obtained from the Ethics Committee of Politecnico di Milano for both protocols (opinions n. 33/2023 and n. 20/2024 for the static and dynamic protocols, respectively).

Subjects with missing or incomplete data were excluded from further analysis. In particular, in this proprietary dataset 25/30 subjects were retained from the static protocol and 5/10 subjects from the dynamic protocol.

In addition, the publicly available UCA-EHAR dataset was also used for external validation and ease of reproducibility of our findings. This dataset includes inertial signals collected from a 6-axis IMU embedded in smart glasses, sampled at 26 Hz, from twenty subjects (12 M and 8 F, age 30.6 ± 12.0 years) performing a comparable set of daily-life activities. Complete details on such dataset composition and acquisition procedures can be found in [10]. For the study on the UCA-EHAR dataset, the following activities were selected: *walking*, *running*, *lying horizontally*, *stairs*, *drinking*, and staying *still* (obtained by merging the original *sitting* and *standing* classes).

The inclusion of UCA-EHAR serves to complement the proprietary dataset by enabling external validation on an independent cohort, different sampling frequency, and a distinct activity set. Together, the two datasets allow for the assessment of OSR behavior across heterogeneous recording conditions that are representative of realistic smart eyewear deployments.

### 3.2. Data Preprocessing

Raw inertial data were segmented using a sliding window approach with a 3 s window and 50% overlap (1.5 s), resulting in 11,231 samples for the proprietary dataset and 13,420 for the UCA-EHAR dataset. Table 3 and Table 4 report the number of samples available for each activity across subjects for the proprietary dataset and the UCA-EHAR dataset, respectively.

As can be noticed, both datasets were not uniformly distributed across activities. No additional signal processing, filtering or normalization were applied, allowing the model to directly learn relevant feature representations from raw sensor data.

### 3.3. Problem Formulation

Let $X\subset R^{\left|T\times d\right|}$ denotes the space of temporal segments extracted from the inertial sensor data, where *T* is the number of samples in each segment and *d* is the number of sensor channels (*d* = 6 in our study involving a three-axis accelerometer and three-axis gyroscope). Each window x∈X is associated with an activity label y ∈ Y. In a conventional closed-set HAR scenario, the learning problem assumes a fixed set of activity classes $Y_{train}\;=\;\{1,\;...,\;C\}$ available during training, and the goal is to learn a classifier:

(1) $$f_{\theta }:X\to Y_{train}$$

parametrized by *θ*, trained on a labelled dataset to minimize the expected classification loss.

In OSR, at test time, the input data may belong to an extended label space $Y_{test}\;=\;Y_{train}\cup \;Y_{unk}$, where $Y_{unk}$ denotes the set of unknown classes not observed during training. The OSR problem requires the system to correctly classify the samples from $Y_{train}$ while detecting when a sample is part of $Y_{unk}$. To this end, we associate each input *x* with an open-set score $S\left(x\right)\in R$ to quantify the degree to which *x* is considered unknown. Higher values of *S*(*x*) indicate a higher likelihood that $x\in Y_{unk}$.

In this work, we consider neural networks as a classifier $f_{\theta }$; thus, *S*(*x*) was derived from the *C*-dimensional logit vector, i.e., the raw outputs $z\left(x\right)=(z_{1}\left(x\right),\;\ldots ,\;z_{C}\left(x\right))\in R^{C}$ of the neural network before the SoftMax layer. All the proposed methods define *S*(*x*) directly on *z*(*x*) while leaving the classifier unchanged. The definition of *S*(*x*) is described in detail in Section 3.5. This design choice reflects our focus on practical OSR solutions that do not require any modification of the backbone training procedure, thus preserving the standard HAR pipeline and facilitating deployment in resource-constrained wearable devices.

In the following, known samples will refer to those belonging to one of the classes available during training, while unknown samples will refer to those originating from classes for which no representative training data exist.

### 3.4. HAR Classifier

**Figure 1:**
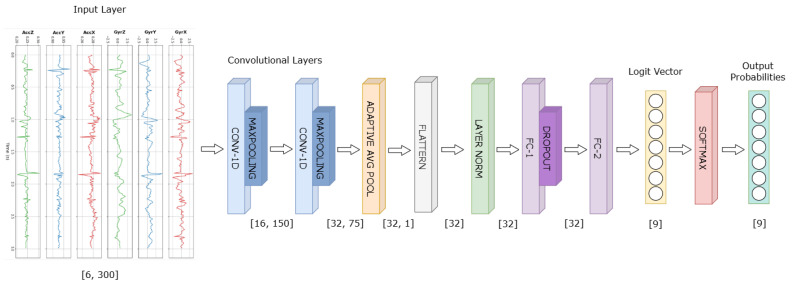
Overview of the proposed CNN architecture for HAR using six-axis IMU windows. The model consists of two convolutional blocks, adaptive average pooling, flattening and layer normalization, followed by a fully connected block producing the class logits. A Softmax activation is applied at inference to obtain output probabilities.

As the effectiveness of OSR strongly depends on the closed-set performance, the first step is to build a highly accurate classifier under the closed-set assumption [33]. The proposed classification backbone consists of a two-layer one-dimensional CNN, designed to prioritize efficiency and ease of deployment while maintaining sufficient discriminative power. The neural network is composed of two convolutional blocks (Conv1D–ReLU–MaxPool) with increasing channel dimensionality (16 → 32) followed by adaptive average pooling to aggregate temporal features and layer normalization to obtain a compact 32-dimensional representation. This feature vector is then passed through a fully connected layer with ReLU activation and dropout regularization and finally projected into the class space through a linear output layer. The resulting logit vector $z\left(x\right)=(z_{1}\left(x\right),\;\ldots ,\;z_{C}\left(x\right))\in R^{C}$ is used both for closed-set classification through $\underset{c}{\hat{y}\left(x\right)=\mathrm{argmax}}\;z_{c}\left(x\right)$ and as the basis for the open-set score *S*(*x*), which is derived by the methods described in the following sections. A representation of the network architecture is reported in Figure 1.

The network was exclusively trained on samples belonging to the known classes $Y_{train}$ using cross-entropy loss with class weights for dataset imbalance. Training was performed with the Adam optimizer using a learning rate of 10^−3^ and weight decay of 10^−5^ over a maximum of 50 epochs. To monitor generalization performance, the samples belonging to the known classes were split into training and validation sets with an 80/20 ratio. A stratified split was employed to preserve the original class distribution in both subsets, preventing biased validation estimates in the presence of class imbalance. Model selection was based on the macro-averaged F1 score on the validation set, which also drives learning rate scheduling and early stopping. All experiments were made reproducible by fixing random seeds across PyTorch v2.5.1, NumPy v2.0.2, and data loaders.

### 3.5. Open-Set Recognition Methods

After training the CNN classifier on the closed-set $Y_{train}$, the open-set score *S*(*x*) was defined on the output logit *z*(*x*). High values of *S*(*x*) indicate that the sample likely belongs to the unknown class set $Y_{unk}$. The following five lightweight OSR scoring strategies were considered, all requiring no modification to the training procedure.

#### 3.5.1. Maximum Logit Score

The first approach is the Maximum Logit Score (MLS), which builds on the intuition that confident predictions correspond to a high activation for one specific class. Given the logit vector *z*(*x*), MLS is defined as the maximum component:

(2) $$MLS(x)\;=\;\underset{c}{\max}\;z_{c}(x).$$

In previous studies [33,34], inputs that do not belong to one of the known classes usually result is a significantly smaller MLS. To maintain a consistent interpretation across all the tested OSR methods, where higher values of the open-set score indicate a higher likelihood of being unknown, the score was defined as the negative of MLS:

(3) $$S_{MLS}\left(x\right)=\;-MLS(x).$$

With this convention, samples from known classes generate lower (and negative) scores, whereas samples from unknown classes yield higher scores, thus aligning the distribution of *S*(*x*) with the general OSR objective.

#### 3.5.2. Energy-Based

As an alternative to MLS, we also considered the energy score [18], which provides a smoother measure of the confidence associated with a logit vector. Given the logits *z(x)*, the energy function is defined as:

(4) $$E\left(x\right)=\;-\;T\cdot \log\sum_{c=1}^{c}\exp\left(\frac{z_{c}\left(x\right)}{T}\right),$$

where *T* > 0 is a temperature parameter. *E*(*x*) tends to be higher for samples of known classes and lower for uncertain predictions. The OSR score is defined directly as the energy:

(5) $$S_{Energy}\left(x\right)=\;E\left(x\right).$$

#### 3.5.3. Nearest-Neighbor Distance Ratio (NNDR)

This method was inspired by the Open-Set Nearest Neighbor (OSNN) classifier proposed by [35]. In their approach, OSR was achieved by extending the traditional nearest-neighbor classifier with the Nearest Neighbor Distance Ratio (NNDR), defined as the ratio between the distance of a test sample to its closest neighbor of the predicted class and the distance to the closest neighbor of a different class. A threshold on this ratio was then used to decide whether the sample should be accepted as known or rejected as unknown. A key advantage of this method is that the OSNN is inherently multiclass, meaning that its efficiency will not be affected as the number of available classes increases.

In our setting, this principle was adapted to the logit space produced by the CNN backbone. Given the predicted class $\hat{y}\left(x\right)=\;\underset{c}{\hat{y}\left(x\right)=\mathrm{argmax}}\;z_{c}\left(x\right)$ and denoting with $Z_{train}^{\left(c\right)}$ the set of training logits corresponding to class *c*, we computed:

(6) $$d_{same}\left(x\right)=\underset{u\in Z_{\mathrm{train}}^{\hat{y}\left(x\right)}}{\min}\left\|z\left(x\right)-u\right\|,$$

(7) $$d_{diff}\left(x\right)=\underset{u\in {Z_{\mathrm{train}}\setminus Z}_{\mathrm{train}}^{\hat{y}\left(x\right)}}{\min}\left\|z\left(x\right)-u\right\|.$$

The NNDR score is defined as:

(8) $$S_{NNDR}\left(x\right)=\;\frac{d_{same}\left(x\right)}{d_{diff}\left(x\right)}.$$

Large values of $S_{NNDR}\left(x\right)$ indicate that the sample lies relatively far from its own class and closer to a different class, suggesting novelty.

#### 3.5.4. Gaussian Mixture Model (GMM)

This approach aims to directly model the distribution of the logit vectors for the known classes using Gaussian Mixture Models (GMMs). For each class $c\in \;Y_{train}$, a single Gaussian component was fitted to the training logits, thus obtaining class-conditional densities $N\left(z(x)\;\right|\mu_{c},\Sigma_{c})$ with prior weights $\pi_{c}$ estimated from the training data. The overall likelihood of a test sample logit *z*(*x*) was then computed as:

(9) $$p_{GMM}\left(z(x)\right)=\;\sum_{c=1}^{C}\pi_{c}N\left(z(x)\;\right|\mu_{c},\Sigma_{c}).$$

The open-set score was defined as the negative log-likelihood:

(10) $$S_{GMM}\left(x\right)=-\log\left(p_{GMM}(z\left(x\right)\right).$$

Intuitively, samples consistent with the distribution of the known classes yield low scores, whereas samples lying outside the support of the known data have small likelihood and thus large scores. In our framework, we restrict each class to a single Gaussian component for efficiency and robustness while maintaining the ability to capture class-specific covariance structures.

#### 3.5.5. Kernel Density Estimation (KDE)

In addition to parametric Gaussian models, a non-parametric approach based on Kernel Density Estimation (KDE) [36] was also explored to model the distribution of logits. For each known class $c\in Y_{train}$, a Gaussian kernel density estimator was fitted to the corresponding training logits $Z_{train}^{\left(c\right)}$. The resulting class-conditional densities were combined with empirical class priors $\pi_{c}$ to define the overall likelihood of a test logit vector:

(11) $$p_{KDE}\left(z\left(x\right)\right)=\sum_{c=1}^{C}\pi_{c}{\hat{p}}_{c}\left(z\left(x\right)\right),$$

where ${\hat{p}}_{c}$ indicates the kernel density estimator for a class *c*. Similarly to the GMM approach, the open-set score is given by the negative log-likelihood:

(12) $$S_{KDE}\left(x\right)=-\log\left(p_{KDE}\left(z(x\right)\right).$$

The intuition is that samples yielding a logit that is consistent with the distribution of known class logits achieve high density under the KDE model, resulting in low scores, whereas unknown samples yield low density and thus high scores. Compared to GMMs, KDE does not impose a parametric form on the class distribution and can therefore capture more flexible logit-space geometries. However, KDE typically requires more training samples than GMM to achieve stable estimates and can be computationally more demanding in high-dimensional settings, in particular when the logit space dimension *C*, corresponding also to the number of classes, is large.

#### 3.5.6. OpenMax

The OpenMax method was originally proposed by Bendale and Boult [14] as one of the first approaches to OSR in deep neural networks. The core idea is to adjust the network’s activations by estimating the probability that a sample lies far from the distribution of known classes in the activation space. Specifically, for each known class, the Mean Activation Vector (MAV) is computed from correctly classified training samples. The distance of a test activation to its class MAV is then modelled using Extreme Value Theory (EVT): a Weibull distribution is fitted to the tail of the distance distribution for each class. At inference time, OpenMax adjusts the most relevant class activations by estimating how likely it is for a sample to be an outlier. The α-highest activations are selected, and for each of them, an outlier probability is computed using the Weibull model. These activations are then reduced in proportion to this probability, and the removed activation mass is reassigned to an additional unknown class, producing an output vector over *C +* 1 categories.

In our framework, this procedure was adopted directly in the logit space of the CNN backbone: per-class MAVs were computed from the training logits, Weibull models were fitted on the extreme distances using the combined Euclidean–cosine metric, and the OpenMax recalibration at inference was applied. The open-set score was defined as the probability assigned to the additional unknown class:

(13) $$S_{OpenMax}\left(x\right)=p_{unk}\left(x\right).$$

This formulation differs from the original use of OpenMax mainly in two aspects: (i) the activation space was restricted to the logit vectors rather than intermediate deep features, thus keeping the backbone unchanged and compatible with our logit-based framework; (ii) the unknown probability was interpreted as a continuous open-set score rather than applying a fixed decision threshold.

These modifications aligned OpenMax with the general scoring-based formulation adopted throughout this work, thus allowing for a consistent comparison with the other considered OSR methods.

### 3.6. Evaluation Strategy

Two complementary evaluation protocols were conducted to assess the model’s abilities, both to classify known activities and to detect previously unseen activities.

To measure how accurately the CNN can distinguish among the target activities, a five-fold subject-wise cross-validation was employed. This protocol enforces strict subject independence between sets, preventing leakage of individual movement patterns. For the proprietary dataset, each fold was composed of 6 held-out subjects for testing (5 from the static protocol and 1 from the dynamic protocol), with the remaining subjects used for training. For the UCA-EHAR dataset, the same five-fold cross-validation was used across subjects. Considering that each subject performed the activity for a similar duration, the proportion of represented activities in each set was balanced. Closed-set performance was reported using accuracy and macro-F1, the latter being more informative for imbalanced HAR scenarios. The results were then aggregated across folds and presented as mean ± standard deviation.

**Figure 2:**
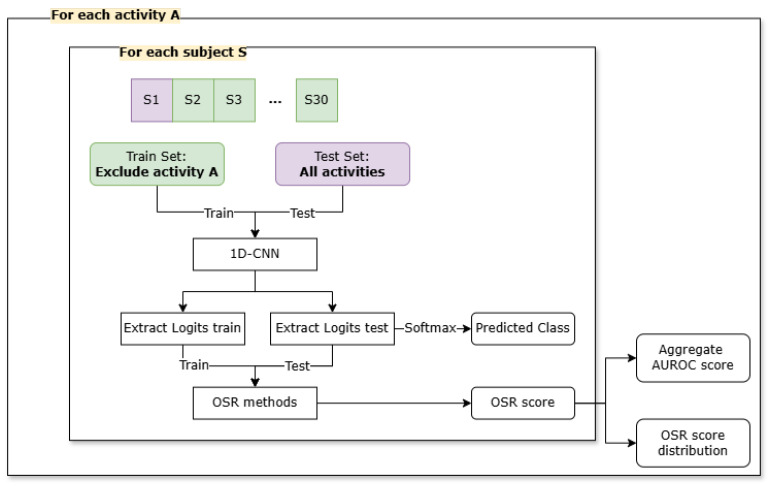
Open-set evaluation through nested leave-one-activity-out (LOAO) and leave-one-subject-out (LOSO) cross-validation.

To evaluate OSR performance, a nested validation scheme was designed combining leave-one-activity-out (LOAO) and leave-one-subject-out (LOSO) cross-validation (Figure 2). At the outer level (i.e., LOAO), one activity class was held out during training and treated as unknown at test time while the classifier was trained on the remaining activities. At the inner level (i.e., LOSO), data from one subject were excluded during training and used exclusively for testing. Here, the set of activities remained the same, but the system was exposed to previously unseen individuals, thereby testing its ability to generalize across subjects.

For each fold in LOAO and LOSO, the base classifier was trained on the corresponding training set, and the logits were collected on the held-out test partition. The open-set scores, defined in the previous section, were calibrated on the training set logits that contained only known classes and then computed for every test instance. As the ground truth labels are available, each test set naturally contains a mixture of known and unknown samples, depending on the held-out condition. This enabled a direct and systematic evaluation of OSR performance across multiple folds.

Discrimination capability was assessed through the area under the receiver operating characteristic curve (AUROC), which summarizes the trade-off between the true positive rate and false positive rate across thresholds.

This evaluation strategy was consistently applied across both the proprietary and the public UCA-EHAR datasets.

## 4. Results

### 4.1. Closed-Set Classification

Table 5 and Table 6 report the closed-set classification performance of the CNN backbone evaluated through 5-fold subject-wise cross-validation on the proprietary and public (UCA-EHAR) datasets, respectively.

Overall, the proposed lightweight model achieved strong recognition performance across all activities except *speaking*, confirming its suitability as a base classifier for subsequent open-set analysis.

On the proprietary dataset, the model reached an average accuracy of 0.917 ± 0.021 and a macro F1-score consistently above 0.85 for most activities. Stationary and head-related activities such as *breathing*, *nodding*, and *shaking* exhibited near-perfect recognition (F1 > 0.96). In contrast, *speaking* showed markedly lower precision and recall (F1 = 0.47 ± 0.17). Dynamic locomotion tasks, such as *walking* and *stairs*, achieved stable yet slightly lower performance (F1 ≈ 0.85–0.88).

On the UCA-EHAR dataset, the classifier achieved a mean accuracy of 0.874 ± 0.035, confirming good generalization. Activities with pronounced motion signatures, such as *still* and *walking*, were recognized with very high reliability (F1 > 0.96), while *running* displayed moderate confusion.

The overall results demonstrated that the CNN backbone attained robust closed-set performance across both datasets, providing a reliable basis for evaluating the OSR methods in the subsequent analyses.

### 4.2. Open-Set Recognition

Table 7 and Table 8 summarize the OSR results obtained under the LOAO protocol for the proprietary and UCA-EHAR datasets, respectively. Each excluded class represented an unseen activity during training, and it was treated as unknown at test time. Reported values correspond to the AUROC, reflecting the system’s ability to distinguish between known and unknown samples.

On the proprietary dataset (Table 7), all methods achieved satisfactory discrimination, with AUROC values generally above 0.80 for most excluded classes. Among the tested approaches, KDE and GMM consistently produced the highest overall performance, reaching AUROC values of 0.90 and 0.91, respectively. OpenMax performed variably: it performed very well for *cycling* (0.91) and *nodding* (0.90) but was less reliable for classes such as *chewing* and *breathing*. The energy-based and MLS baselines achieved competitive AUROC values.

On the UCA-EHAR dataset (Table 8), performance patterns were broadly consistent though with slightly larger variability across activities. As in the proprietary dataset, KDE and GMM achieved very good results, particularly for *lying* (>0.95) and *still* (≈0.90), both characterized by stable and easily separable motion profiles. More challenging conditions emerged for *running*, where all methods showed lower discrimination. Notably, OpenMax yielded the highest AUROC (0.82). In contrast, energy-based scoring showed the largest variability, underperforming especially on highly dynamic activities.

Overall, the results demonstrate that OSR from head-mounted inertial data is feasible even with lightweight post hoc methods, with density-based approaches achieving the best performances.

## 5. Discussion

Our results showed that, with the proposed framework, OSR from head-mounted inertial data was both feasible and effective using lightweight, post hoc scoring strategies.

The CNN backbone provided a strong closed-set baseline, achieving accuracy above 90% on both datasets. The network achieved near-perfect recognition for static or head-centric activities such as *breathing*, *nodding*, and *shaking*, confirming that head-mounted IMUs can capture fine-grained motion patterns associated with subtle head and upper-body movements. The most challenging class was *speaking*, which exhibited both a low average F1 score and high variability across folds. This drop can be attributed to both confusion with similar activities and data scarcity. Indeed, *speaking* was included only in the dynamic protocol; thus, each training fold contained recordings from four subjects and a total duration of approximately 1 min per subject, considerably shorter than other activities (e.g., 9 min of *walking* or 15 min of *cycling* per subject) or static activities (20 subjects in the training fold).

The open-set results revealed that performance is influenced by the choice of the excluded activity, confirming that OSR performance is class-dependent, as some classes are easier to separate from others. For example, in experiments involving the proprietary dataset, when *breathing* was excluded and treated as unknown, all methods failed to effectively separate it from the known activities. This outcome suggests that *breathing* acts as a baseline activity, characterized by low-motion patterns that overlap with many other behaviors. Consequently, omitting it from the training set deprives the model of a reference for “neutral” motion, impairing novelty detection. This behavior was not observed on the UCA-EHAR dataset, likely because even when *still* was excluded, another low-dynamic activity (*lying*) remained in the training set, effectively serving as a substitute baseline. These findings highlight that the availability of at least one baseline or low-motion class during training appears crucial for stable OSR in HAR when using head-mounted sensors.

Additional class-specific failure modes emerge for highly dynamic activities. In the UCA-EHAR dataset, when *running* is treated as an unknown class, it is often confused with known activities such as *walking* and *stairs* (which also exhibit large acceleration values), leading to confident but incorrect predictions, particularly for confidence-based scoring methods such as the energy score. In these cases, the learned representations for unknown samples fall in regions in the logit space characterized by other activities, resulting in poor open-set discrimination. Overall, these observations indicate that accurate recognition of unknown activities is achieved when known activity classes are sufficiently well separated in the learned representation space, and when baseline or low-motion activities are available during training.

Across both datasets, density-based approaches (KDE, GMM) consistently achieved the most stable AUROC values, indicating that modelling the logit distribution of known classes provides a reliable boundary between familiar and unseen activities. OpenMax also performed competitively, particularly on UCA-EHAR. Simpler confidence-based methods (MLS, energy) are also promising as computational efficient alternatives.

Taken together, the closed-set and open-set results highlight a relationship between base classification quality and open-set behavior. While in computer vision it is widely acknowledged that an accurate closed-set classifier is needed to perform open-set recognition [33], in our experiments we discovered that accurate closed-set performance does not guarantee robust open-set recognition. In fact, separation boundaries established by the open-set classifier are influenced by the location of all the known activity classes in the latent space. For example, a class that presents very distinctive patterns (e.g., *speaking*, which is an activity where the head is mostly still) but that is poorly represented in the training set may still be easily detected as unknown when excluded from the known set, even if its closed-set classification performance is low due to class imbalance. However, when weak or highly variable classes remain part of the known set, they can reduce the stability and separability of learned representations, increasing overlap in the logit space and negatively impacting open-set discrimination. Overall, our results confirm that open-set recognition performance depends not only on closed-set accuracy but also on the stability and separability of learned representations across classes.

Beyond the quantitative performance of individual methods, the results highlight domain-specific challenges that distinguish IMU-based HAR from image recognition, a field where OSR techniques have been largely explored in the literature. Inertial data are multivariate temporal signals characterized by strong inter-axis coupling, substantial inter-subject variability, and overlap between semantically related activities, such as walking, running, and stair climbing. On top of that, user-specific traits suggest that HAR models need to be easy to be personalized. The customary operating conditions of HAR and image classification are also completely different, as the former refer to embedded devices and the latter also to more powerful computing devices. Training datasets are also typically of different sizes. All in all, these characteristics result in a substantial difference in DL models for the two domains and underline the importance of investigating lightweight and portable OSR solutions in HAR.

An important aspect for practical deployment on wearable platforms concerns the computational and memory costs associated with the different OSR strategies. The considered OSR methods exhibit distinct inference time complexities and storage requirements. Confidence-based approaches, such as MLS and energy scoring, operate directly on the logit output and introduce negligible computational overhead, requiring only simple arithmetic operations and no additional stored parameters beyond the classifier weights. Distance- and non-parametric density-based methods require storing training logits and performing comparisons with multiple samples at inference time; in particular, NNDR and KDE incur computational costs and memory usage that scale with the number of stored training examples. In contrast, GMM-based scoring models each known class using a small, fixed set of parameters (mean and covariance), leading to inference complexity that scales with the number of training classes. OpenMax introduces an additional recalibration step based on Extreme Value Theory, involving distance computations and Weibull evaluations for the most activated classes, with both computation and memory linearly scaling with the number of known classes. Overall, while all evaluated methods remain lightweight compared to retraining-based or generative OSR approaches, confidence-based and parametric density-based strategies exhibit the lowest computational and memory overhead, making them particularly suitable for resource-constrained wearable HAR systems.

A further practical aspect for deployment concerns the selection of the decision threshold used to separate known from unknown samples. In this work, thresholds are not fixed a priori but implicitly explored through the AUROC metric, allowing a fair comparison across OSR methods to identify the one yielding the best separation among known and unknown classes (repeated in an LOAO fashion). In real-world wearable scenarios, however, the operating threshold must be selected to control the rate of false rejections of known activities, according to the application requirements. For instance, safety-critical applications may favor conservative thresholds that reduce the risk of accepting unknown activities as known, while operating at low false alarm rates is key in non-critical continuous monitoring scenarios. Importantly, since all considered OSR methods produce continuous scores defined on the logit space, threshold calibration can be performed post hoc using a small validation set representative of the deployment context without retraining the classifier.

## 6. Limitations and Future Directions

The results presented in this work indicate that reliable OSR performance can be achieved from head-mounted inertial data using lightweight, post hoc scoring methods. Although the open-set decision process does not explicitly incorporate temporal dependencies or inter-axis dynamics, these characteristics are captured at the representation level by the neural classifier trained on raw time-series data from a multichannel IMU. This suggests that a substantial portion of the temporal and multivariate information relevant for novelty detection is already preserved in the classifier outputs and can be effectively exploited by simple scoring rules. Recent studies [37,38,39] on OSR for time-series data have shown that explicitly modelling temporal and multivariate structure within the open-set mechanism, through approaches such as contrastive learning across time or frequency domains, reconstruction-based objectives, or time-series-specific similarity measures, can further improve novelty detection. However, these methods typically introduce additional model components, training objectives, or computational overhead, representing a less efficient and practical solution than the lightweight OSR module cascaded to the latent representation.

Building on these observations, a promising direction for future work is to investigate how temporal-aware open-set mechanisms could be integrated with lightweight post hoc scoring strategies with the aim of improving robustness while preserving the efficiency and embeddability required by wearable HAR systems.

A further limitation of this study concerns the scale and distribution of the available data. The proprietary dataset includes a limited number of subjects, particularly for the dynamic protocol, and both the proprietary and public datasets exhibit class imbalance. These characteristics reflect realistic constraints of wearable IMU data collection, where acquisition protocols, user compliance, and activity frequency are inherently heterogeneous. These factors may affect the statistical characterization of class-conditional logit distributions and, in turn, influence the stability of OSR for underrepresented or highly variable activities. Future work will explore the extension of this analysis to larger and more diverse cohorts, as well as strategies to improve robustness to class imbalance in open-set HAR.

## 7. Conclusions

In summary, this study demonstrates that head-mounted IMU data can effectively support open-set HAR when coupled with lightweight, post hoc scoring strategies. The results show that open-set performance is not solely determined by the choice of the scoring method but critically depends on the composition and quality of the known activity set, particularly the presence of baseline or low-motion activities and sufficient representation for each class. Beyond benchmarking individual techniques, these findings provide practical insights into the conditions under which open-set HAR systems can be reliably deployed, offering concrete design guidelines for building robust and resource-efficient wearable systems capable of handling previously unseen activities in real-world scenarios.

## Figures and Tables

**Table 1:** Description and duration of each activity included in the static protocol acquisitions.

Activity	Description	Duration
*Chewing*	Chew gum	30 s
*Drinking*	Drink a glass of water in sips	1 min
*Nodding*	Nod with the head	30 s
*Shaking*	Shake the head	30 s
*Breathing*	Remain still in a seated position	3 min

**Table 2:** Description and duration of each activity included in the dynamic protocol acquisitions.

Activity	Description	Duration
*Speaking*	Speak freely	1 min
*Walking*	Walk on the treadmill at increasing speed	9 min
*Cycling*	Cycle at increasing load	15 min
*Stairs*	Walk ascending and descending stairs	2 min

**Table 3:** Number of samples obtained per activity with a sliding window of 3 s and 50% overlap on the proprietary dataset (static and dynamic protocol). Values are reported as median [25 percentile; 75 percentile] across subjects.

Activity	Number of Samples *(Median [25 Percentile; 75 Percentile])*
*Chewing*	26 [25; 28]
*Drinking*	33 [23; 39]
*Nodding*	19 [18; 20]
*Shaking*	19 [19; 20]
*Breathing*	124 [123; 125]
*Speaking*	42 [39; 43]
*Walking*	381 [377; 390]
*Cycling*	635 [629; 662]
*Stairs*	79 [74; 80]

**Table 4:** Number of samples obtained per activity with a sliding window of 3 s and 50% overlap on UCA-EHAR dataset. Values are reported as median [25 percentile; 75 percentile] across subjects.

Activity	Number of Samples *(Median [25 Percentile; 75 Percentile])*
*Drinking*	40 [32; 101]
*Lying*	79 [59; 94]
*Running*	104 [79; 119]
*Stairs*	112 [79; 152]
*Still*	192 [162; 216]
*Walking*	149 [108; 181]

**Table 5:** Closed-set classification results (5-fold subject-wise cross-validation) on the proprietary dataset. Results are reported as mean ± standard deviation across folds.

Class	Precision	Recall	F1-Score
*Chewing*	0.944 ± 0.065	0.926 ± 0.024	0.934 ± 0.039
*Cycling*	0.958 ± 0.056	0.900 ± 0.115	0.922 ± 0.060
*Drinking*	0.825 ± 0.054	0.874 ± 0.056	0.847 ± 0.039
*Breathing*	0.991 ± 0.008	0.962 ± 0.029	0.976 ± 0.018
*Nodding*	0.945 ± 0.069	0.995 ± 0.007	0.967 ± 0.037
*Shaking*	0.979 ± 0.017	0.998 ± 0.004	0.989 ± 0.008
*Speaking*	0.441 ± 0.169	0.565 ± 0.235	0.467 ± 0.165
*Stairs*	0.726 ± 0.128	0.942 ± 0.023	0.815 ± 0.083
*Walking*	0.915 ± 0.104	0.873 ± 0.108	0.883 ± 0.052
Accuracy	0.917 ± 0.021		

**Table 6:** Closed-set classification results (5-fold subject-wise cross-validation) on the UCA-EHAR dataset. Results are reported as mean ± standard deviation across folds.

Class	Precision	Recall	F1-Score
*Drinking*	0.821 ± 0.152	0.926 ± 0.024	0.934 ± 0.039
*Lying*	0.992 ± 0.006	0.900 ± 0.115	0.922 ± 0.060
*Running*	0.855 ± 0.131	0.874 ± 0.056	0.847 ± 0.039
*Stairs*	0.833 ± 0.048	0.962 ± 0.029	0.976 ± 0.018
*Still*	0.964 ± 0.016	0.995 ± 0.007	0.967 ± 0.037
*Walking*	0.807 ± 0.063	0.998 ± 0.004	0.989 ± 0.008
Accuracy	0.874 ± 0.035		

**Table 7:** Open-set recognition results (AUROC) under leave-one-activity-out protocol on the proprietary dataset. Each excluded class represents an unseen activity during training (values < 0.7 in italic, values >= 0.9 in bold).

Excluded Class	Max Logit	Energy	NNDR	GMM	KDE	OpenMax
*Chewing*	0.798	0.838	0.805	0.851	0.871	0.638
*Cycling*	**0.900**	0.814	0.798	0.884	0.881	**0.912**
*Drinking*	0.850	0.876	0.858	0.887	**0.930**	0.786
*Breathing*	0.716	*0.550*	0.804	*0.678*	*0.675*	*0.657*
*Nodding*	**0.921**	0.778	0.870	**0.902**	**0.914**	**0.900**
*Shaking*	**0.967**	**0.936**	0.898	0.843	**0.913**	0.835
*Speaking*	0.841	0.773	0.810	0.883	**0.907**	0.796
*Stairs*	**0.927**	0.730	0.848	**0.900**	0.878	0.874
*Walking*	0.853	0.807	0.817	0.895	0.853	0.845

**Table 8:** Open-set recognition results (AUROC) under leave-one-activity-out protocol on the UCA-EHAR dataset. Each excluded class represents an unseen activity during training (values < 0.7 in italic, values >= 0.9 in bold).

Excluded Class	Max Logit	Energy	NNDR	GMM	KDE	OpenMax
*Drinking*	0.758	*0.697*	0.766	0.765	0.730	0.835
*Lying*	**0.956**	**0.966**	0.843	**0.957**	**0.962**	**0.923**
*Running*	*0.518*	*0.365*	0.724	*0.668*	*0.637*	*0.683*
*Still*	0.893	0.786	0.794	0.893	0.897	**0.904**
*Stairs*	0.751	*0.679*	0.745	0.798	0.814	0.785
*Walking*	0.760	0.706	0.808	0.807	0.827	0.818

## Data Availability

The data presented in this study can be made available following reasonable request to the corresponding author.

## Abbreviations

The following abbreviations are used in this manuscript:

HAR	Human Activity Recognition
OSR	Open-Set Recognition
IMU	Inertial Measurement Unit
CNN	Convolutional Neural Network
MLS	Maximum Logit Score
NNDR	Nearest-Neighbor Distance Ratio
GMM	Gaussian Mixture Model
KDE	Kernel Density Estimation
LOAO	Leave-One-Activity-Out
LOSO	Leave-One-Subject-Out
AUROC	Area Under the Receiver Operating Characteristic curve

## References

[1] Bulling A., Blanke U., Schiele B. (2014). A Tutorial on Human Activity Recognition Using Body-Worn Inertial Sensors. ACM Comput. Surv..

[2] Chen K., Zhang D., Yao L., Guo B., Yu Z., Liu Y. (2021). Deep learning for sensor-based human activity recognition: Overview, challenges and opportunities. ACM Comput. Surv..

[3] Lara O.D., Labrador M.A. (2013). A Survey on Human Activity Recognition Using Wearable Sensors. IEEE Commun. Surv. Tutor..

[4] Haresamudram H., Ian Tang C., Lukowicz P., Kaiserslautern-Landau R., Thomas Plötz G. (2025). Past, Present, and Future of Sensor-Based Human Activity Recognition Using Wearables: A Surveying Tutorial on a Still Challenging Task. Proc. ACM Interact. Mob. Wearable Ubiquitous Technol..

[5] Sun J., Dong Q. (2023). A survey on open-set image recognition. arXiv.

[6] Halász A.P., Al Hemeary N., Daubner L.S., Juhász J., Zsedrovits T., Tornai K. (2025). Adapting a Previously Proposed Open-Set Recognition Method for Time-Series Data: A Biometric User Identification Case Study. Electronics.

[7] Boborzi L., Decker J., Rezaei R., Schniepp R., Wuehr M. (2024). Human Activity Recognition in a Free-Living Environment Using an Ear-Worn Motion Sensor. Sensors.

[8] Stankoski S., Sazdov B., Broulidakis J., Kiprijanovska I., Sofronievski B., Cox S., Gjoreski M., Archer J., Nduka C., Gjoreski H. (2023). Recognizing Activities of Daily Living Using Multi-Sensor Smart Glasses. medRxiv.

[9] Ho J., Wang C.M. (2016). User-Centric and Real-Time Activity Recognition Using Smart Glasses. Proceedings of the Green, Pervasive, and Cloud Computing.

[10] Novac P.E., Pegatoquet A., Miramond B., Caquineau C. (2022). UCA-EHAR: A Dataset for Human Activity Recognition with Embedded AI on Smart Glasses. Appl. Sci..

[11] Mekruksavanich S., Jantawong P., Jitpattanakul A. Deep Learning Approaches for HAR of Daily Living Activities Using IMU Sensors in Smart Glasses. Proceedings of the 2023 Joint International Conference on Digital Arts, Media and Technology with ECTI Northern Section Conference on Electrical, Electronics, Computer and Telecommunications Engineering (ECTI DAMT & NCON).

[12] Sloan W., Wallace B., Goubran R., Sveistrup H. (2025). Cross-Body Transfer Learning for Human Activity Recognition. Proceedings of the 2025 IEEE International Instrumentation and Measurement Technology Conference (I2MTC), Ottawa, ON, Canada, 19–22 May 2025.

[13] Lee M., Kim S.B. (2022). Sensor-based open-set human activity recognition using representation learning with mixup triplets. IEEE Access.

[14] Sousa Lima W., Bragança H., Souto E. (2020). NOHAR—Novelty discrete data stream for human activity recognition based on smartphones with inertial sensors. Expert Syst. Appl..

[15] Roy D., Komini V., Girdzijauskas S. Out-of-Distribution in Human Activity Recognition. Proceedings of the 2022 Swedish Artificial Intelligence Society Workshop (SAIS).

[16] Roy D., Komini V., Girdzijauskas S. (2023). Classifying Falls Using Out-of-Distribution Detection in Human Activity Recognition. AI Commun..

[17] Kim H., Lee D. (2024). Self-supervised New Activity Detection in Sensor-based Smart Environments. arXiv.

[18] Boyer P., Burns D., Whyne C. (2021). Out-of-Distribution Detection of Human Activity Recognition with Smartwatch Inertial Sensors. Sensors.

[19] De Carolis B., Ferilli S., Mallardi G. (2014). Learning and Recognizing Routines and Activities in SOFiA. Ambient Intelligence.

[20] Scheirer W.J., de Rezende Rocha A., Sapkota A., Boult T.E. (2013). Toward Open Set Recognition. IEEE Trans. Pattern Anal. Mach. Intell..

[21] Bendale A., Boult T.E. (2016). Towards Open Set Deep Networks. IEEE Conference on Computer Vision and Pattern Recognition.

[22] Hendrycks D., Gimpel K. (2016). A Baseline for Detecting Misclassified and Out-of-Distribution Examples in Neural Networks. arXiv.

[23] Nguyen A., Yosinski J., Clune J. (2015). Deep neural networks are easily fooled: High confidence predictions for un-recognizable images. 2015 IEEE Conference on Computer Vision and Pattern Recognition (CVPR ’15).

[24] Dai W., Diao W., Sun X., Zhang Y., Zhao L., Li J., Fu K. (2021). CAMV: Class activation mapping value towards open set fine-grained recognition. IEEE Access.

[25] Liu W., Wang X., Owens J., Li Y. (2020). Energy-Based Out-of-Distribution Detection. Proceedings of the Advances in Neural Information Processing Systems.

[26] Liang S., Li Y., Srikant R. Enhancing the Reliability of Out-of-distribution Image Detection in Neural Networks. Proceedings of the International Conference on Learning Representations.

[27] Guo C., Pleiss G., Sun Y., Weinberger K.Q. On Calibration of Modern Neural Networks. Proceedings of the International Conference on Machine Learning.

[28] Pereyra G., Tucker G., Chorowski J., Kaiser Ł., Hinton G. Regularizing Neural Networks by Penalizing Confident Output Distributions. Proceedings of the International Conference on Learning Representations.

[29] Oza P., Patel V.M. (2019). C2AE: Class Conditioned Auto-Encoder for Open-Set Recognition. Proceedings of the 2019 IEEE/CVF Conference on Computer Vision and Pattern Recognition (CVPR), Long Beach, CA, USA, 15–20 June 2019.

[30] Lee K., Lee H., Lee K., Shin J. (2017). Training confidence-calibrated classifiers for detecting out-of-distribution samples. arXiv.

[31] Ge Z., Demyanov S., Garnavi R. (2017). Generative OpenMax for multi-class open set classification. Proceedings of the British Machine Vision Conference (BMVC).

[32] Crupi I., Scandelli A., Giudici A., Gervasoni G., Trojaniello D., Villa F. (2025). Multi-sensor smart eyewear for biomarkers acquisition. IEEE Sens..

[33] Vaze S., Han K., Vedaldi A., Zisserman A. Open-Set Recognition: A Good Closed-Set Classifier Is All You Need?. Proceedings of the International Conference on Learning Representations (ICLR).

[34] Hsu Y.-C., Shen Y., Jin H., Kira Z. (2020). Generalized ODIN: Detecting Out-of-Distribution Image Without Learning from Out-of-Distribution Data. 2020 IEEE/CVF Conference on Computer Vision and Pattern Recognition (CVPR).

[35] Mendes Júnior P.R., de Souza R.M., de Werneck R.O., Stein B.V., Pazinato D.V., de Almeida W.R., B Penatti O.A., da Torres R.S., Rocha A., Blockeel Pedro R Mendes Júnior H.B. (2016). Nearest Neighbors Distance Ratio Open-Set Classifier. Mach. Learn..

[36] Erdil E., Trippe B.L., Haque A., Brockschmidt M., Nowozin S. (2020). Unsupervised Out-of-Distribution Detection Using Kernel Density Estimation. arXiv.

[37] Marques L.d.M., Lazzaretti A.E., Lopes H.S. (2024). Blending Ensemble Applied to Open-Set Recognition for Time Series Classification. Learn. Nonlinear Models.

[38] Hu S., Zhao X., Hu S., Gao X. (2025). Fusion-OSR: Cross-Domain Contrastive Learning with Weibull Calibration for Time Series Open Set Recognition. Proceedings of the IEEE International Conference on Acoustics, Speech and Signal Processing (ICASSP 2025), Hyderabad, India, 6–11 April 2025.

[39] Oh H., Kim S.B. (2022). Multivariate Time Series Open-Set Recognition Using Multi-Feature Extraction and Reconstruction. IEEE Access.

